# The child dental care reform in Israel – service uptake from 2011 to 2022

**DOI:** 10.1186/s13584-024-00630-y

**Published:** 2024-08-29

**Authors:** Hagit Domb Herman, Hazav Dadosh, Dan Dekel, David Yellon, Shlomo Paul Zusman, Lena Natapov

**Affiliations:** Division of Dental Health, Ministry of Health, Jerusalem, Israel

**Keywords:** Universal health coverage, Dental care, Children, Reform, Public health policy

## Abstract

**Background:**

The 2010 Child Dental Care Reform of the National Health Insurance Law marked a turning point in the Israeli oral healthcare system by establishing Universal Health Coverage of dental care for children. Initially, the reform included children up to age 8 and gradually expanded to age 18 in 2019. The basket of services includes preventive and restorative treatments provided by the four Health Maintenance Organizations (HMO). The aim of this study was to examine the uptake of child dental services during the first decade of the reform.

**Methods:**

A retrospective analysis was conducted to determine the treatment uptake, type and amount of the services delivered based on annual service utilization reports submitted by the HMOs to the Ministry of Health in the years 2011–2022.

**Results:**

The number of insured children increased from 1,546,857 in 2011 to 3,178,238 in 2022. The uptake of dental services gradually increased during the study period with a slight decrease in 2020. The percentage of children who used the services gradually increased from 8 to 33%, with the incremental inclusion of additional age groups. From 2012 onwards the most common treatments provided were preventive, however the single most common treatment was dental restoration. In 2022 35% of the population of Israel was under the age of 18. Out of these, about a third received dental treatment via the HMOs. This is a significant achievement, since before the reform all treatments were paid out-of-pocket. After a short period of increasing uptake, a stable service utilization pattern was evident that can indicate better public awareness and service acceptance.

**Conclusion:**

Although this is a reasonable uptake, additional efforts are required to increase the number of children receiving dental care within the public insurance. Such an effort can be part of a multi-disciplinary approach, in which pediatricians and public health nurses can play a vital role in dental caries prevention, enhancement of awareness and service utilization.

## Background

Oral health has an integral role in the overall health and quality of life of an individual. Oral health status affects physical, psychological, emotional and social well-being [1]. Disparities in access to oral health care can cause oral health neglect, especially in individuals from socio-economic disadvantaged backgrounds. Universal health coverage (UHC) facilitates equitable access to health services and address health disparities. Integration of oral health into universal health coverage reflects a commitment to comprehensive medical care, fostering healthier populations and communities [2].

In order to provide equitable health coverage to the Israeli population, the Israeli National Health Insurance Law (NHIL) was enacted in 1994. The NHIL established universal health coverage with a basket of health services provided by four Health Maintenance Organizations (HMOs). However, dental treatment was not included in the basket of services in 1994 except for specific conditions or patient groups, e.g. oncology patients, maxillo-facial surgery in cases of trauma etc.

Almost all child dental services at that time were privately funded, except for the School Dental Service (SDS) operated by local authorities that offered basic dental care to primary school students. Caries prevalence among children, including the level of untreated disease, was high. Thus, a survey conducted by Zadik et al. in 1989 [3] found the Decayed, Missing, Filled Teeth (dmft/DMFT) scores of 5 and 12-year-olds to be 2.72 and 2.99 respectively. Furthermore, a 2002 national Israeli survey by Zusman et al. [4] found that only 46% of 12-year-old children were caries free. A later survey in 2007 conducted as part of the SDS, found that among 5-year-olds, the average dmft score was 3.31 with only 35.3% of the children caries free [5].

Higher caries levels were found in children from lower socio-economic or socially disadvantaged backgrounds. The high costs of dental care in Israel mainly affected poorer and socially disadvantaged groups. Thus, a survey by Shahrabani among mothers of 5-18-year-olds children found that one of the main reasons for the lack of routine dental care was the financial costs [6].

The Child Dental Care Reform (CDCR) in the framework of NHIL was enacted in Israel in 2010 with the aim of reducing these disparities and providing affordable dental care to all children. It consisted of including preventive and restorative treatment for children in the basket of services provided by the NHIL. Criteria for the accessibility and availability of dental treatments for children [7] were established by the Ministry of Health (MOH), beginning with the inclusion of preventive and restorative dental treatment for children from birth to 8. The eligibility age gradually increased to 12 in 2012, 14 in 2016 and 18 in 2019. Dental treatment was provided free of charge or with a small co-payment by the four HMOs in dental clinics countrywide.

The purpose of this study was to evaluate the utilization patterns of dental services within the framework of the reform in its first decade, between the years 2011–2022.

## Methods

This retrospective study analyzed annual data submitted by the four HMOs to the MOH, regarding dental services utilization within the NHIL reform between 2011 and 2022. The data was anonymous and included the number of eligible and treated children by age group, types and number of treatments provided.

The treatments provided were divided into two groups: preventive (examination & treatment plan, hygienist appointment, topical fluoride application, oral hygiene instructions and fissure sealants) and restorative (dental restoration, pulp treatment, dental post, pediatric dental crown, extraction, space maintainer, and emergency care). Utilization was defined as at least one treatment code per individual.

The annual size of the children population (0–18-year-olds, prior to 2019) was obtained from the Central Bureau of Statistics [8].

### Ethical approval

The study was approved by the Ministry of Health Ethics Committee (MOH 119–2023). All methods were performed in accordance with the ethical standards as laid down in the Declaration of Helsinki and its later amendments or comparable ethical standards. Written informed consent from participants or if participants are under 16, from a parent and/or legal guardian was waived.

## Results

### Coverage

**Fig. 1:**
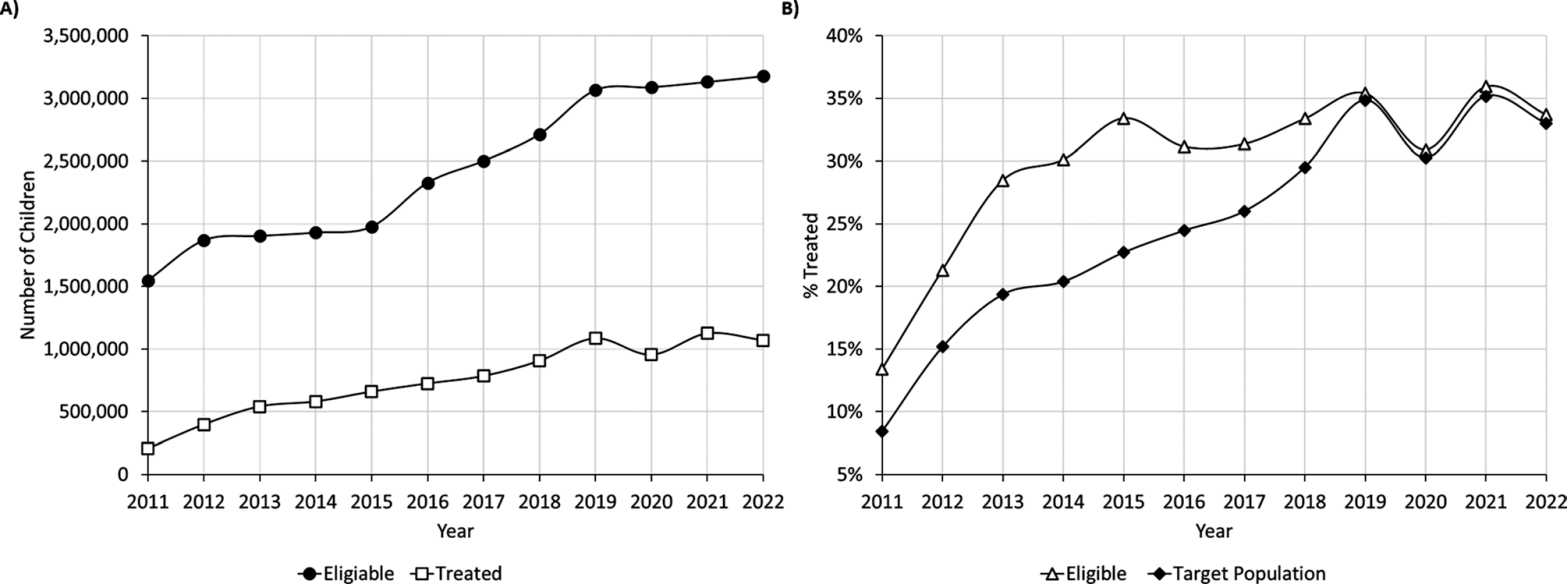
Coverage & uptake within the reform framework (**A**) Absolute number of eligible children and children treated (**B**) Percentage of children treated out of the eligible and out of the total target population of children

From 2011 to 2019, a significant increase in both the number of eligible and treated children was evident. The number of eligible children increased from 1,546,857 in 2011 to 3,178,238 in 2022, in line with the inclusion of additional age groups. In 2011, 207,085 children received dental treatment, with the number growing to 1,071,017 in 2022 (Fig. 1A). Out of the total target population of children from birth up to age 18, the percentage of children treated within the reform framework increased four times during the study period, from 8% in 2011 to 33% in 2022. The service uptake rate significantly increased during the first 4 years of the reform and from 2014 onwards fluctuated between 30 and 35% (Fig. 1B). (*p* < 0.001)

### Treatments provided within the reform framework

**Fig. 2:**
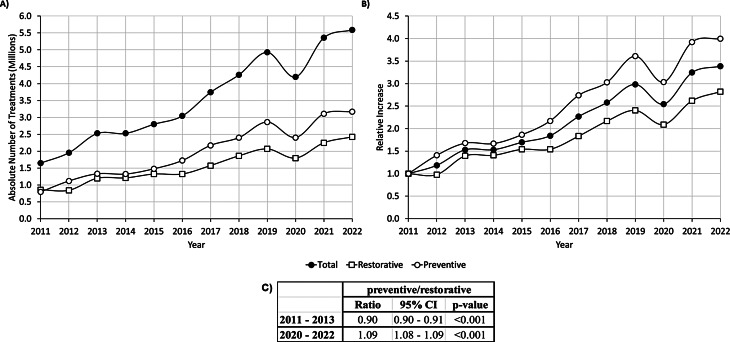
Preventive, and restorative treatments provided within the reform framework. (**A**) Absolute number of total, preventive, and restorative treatments by year. (**B**) Relative increase in total, preventive, and restorative number of treatments by year. (**C**) Comparing between the end of the decade (2020–2022) and the start of the reform (2011–2013)

The total number of treatments increased from 1,651,906 treatments in 2011 to 5,594,483 in 2022. The majority of treatments provided were preventive, with a fourfold increase from 793,107 in 2011 to 3,169,787 in 2022. The relative increase for restorative care was 2.8 fold, from 858,799 to 2,424,696 respectively (Fig. 2A and B).

**Fig. 3:**
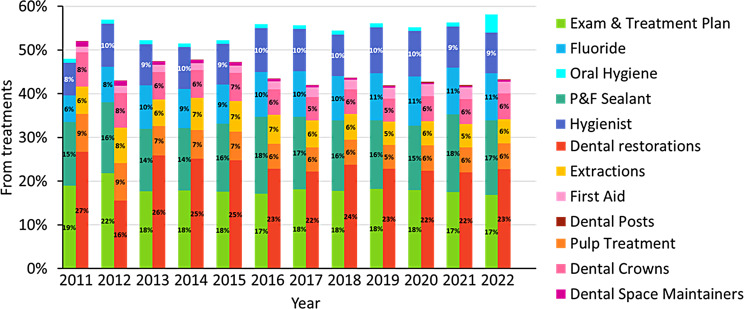
Percentage of different treatments administered under the NHIL dental care reform (percentages of total treatment in each year)

From the start of the reform (2011–2013) to the end of the decade (2020–2022) the percentage of preventive care increased from 53 to 57% (*p* < 0/001), while the percentage of restorative care decreased from 47 to 43% (*p* < 0.001). This changed the ratio between the preventive and restorative care substantially as can be seen in Fig. 3.

Average number of treatments per child during the study period decreased from 7.98 in 2011, to 5.22 in 2022.

### Treatment type provided

Further analysis of the treatment type (by code) showed that from 2012 and onwards, the majority of treatments administered under the NHIL dental care reform were preventive (Fig. 3). In 2022, 58% of administered treatments were preventive.

The most common preventive treatments were dental examination and fissure sealant, each of them constituting 17% of the total in 2022. Among restorative care, dental restorations were the most common treatment, 23% of the total in 2022. (*p* < 0.001)

From 2013 onwards, no significant changes in treatment pattern were observed with the progression of the reform and the gradual inclusion of additional age groups. The frequency of administered treatments remained more or less stable even with the outbreak of COVID-19 in 2020.

### Age-specific treatment utilization rate

The initial age-specific treatment utilization rate was compared to the similar rate in 2022 (Table 1**).** The data presents higher initial utilization in older ages, which were included later during the reform.

The increase in utilization rate is evident in almost all age groups, especially among 5–12 years-old children. The highest increase, of more than 3 times, in the utilization rate during the first decade of the reform occurred in the 9-10-year-olds group, which is resulted in 52.19% and 49.65% of care utilization respectively.

The 6-9-year-olds age group is the most treated. More than half of all children in this age group received dental treatment within the reform, with the percentage of uptake ranging from 50.26 to 53.81%.

**Table 1:** Utilization rate by age group (comparison between inclusion year and 2022)

Age	Inclusion year*	Participation rate		Fold change	CI 95%	*p*-value
		Inclusion	2022			
1	2010	0.20%	0.26%	1.33	1.22 - 1.45	<0.001
2	2010	1.82%	3.51%	1.92	1.88 - 1.97	<0.001
3	2010	5.41%	10.98%	2.03	2.00 - 2.06	<0.001
4	2010	11.85%	26.89%	2.27	2.25 - 2.29	<0.001
5	2010	17.98%	44.53%	2.48	2.46-2.49	<0.001
6	2010	22.16%	50,26%	2.27	2.25 - 2.28	<0.001
7	2010	24.71%	53.81%	2.18	2.16-2.19	<0.001
8	2010	24.45%	53.73%	2.20	2.18-2.21	<0.001
9	2011	14.56%	52.19%	3.58	3.56-3.61	<0.001
10	2011	13.67%	49.65%	3.63	3.61 3.66	<0.001
11	2012	18.38%	46.19%	2.51	2.50 2.53	<0.001
12	2012	20.17%	44.23%	2.19	2.18 2.21	<0.001
13	2016	38.55%	42.12%	1.09	1.08 1.10	<0.001
14	2016	35.02%	39.68%	1.13	1.12 1.14	<0.001
15	2017	36.04%	38.94%	1.08	1.07 1.09	<0.001
16	2018	34.61%	36.74%	1.06	1.05 1.07	<0.001
17	2019	35.29%	34.51%	0.98	0.97 0.99	<0.001
18	2019	36.55%	37.05%	1.01	1.01 1.02	<0.001

*p* < 0.001

* First data available

## Discussion

Prior to the NHIL dental care reform in 2010, most dental treatments for children were provided on a private basis. A high level of dental morbidity and a low level of treatment coverage along with high, mostly out of pocket costs of dental care, characterized the pre-reform period. There were signs of a market failure, which were more prominent amongst children of lower socio-economic groups that presented with high prevalence rates of untreated disease [9]. The reform enabled equitable access to oral care for children by including dental treatments in the basket of services universally covered by the NHIL. By 2019, gradual expansion of eligibility criteria enabled the inclusion of over 3 million children up to 18-year-olds. This represents a major milestone, as more than a third of Israel’s population are now entitled to universal oral healthcare.

As additional age groups were included into the universal coverage, public awareness and acceptance of the reform increased. This was supported by several campaigns led by the MOH, and resulted in constant increase in dental treatment utilization during the study period. A slight decrease in utilization rate was evident was during 2020, probably due to the Covid-19 pandemic which affected routine dental care delivery in Israel [10].

During the study period, the percentage of children who utilized the services increased significantly in all age groups, especially among 12-year-olds and younger children that were included earlier in the reform. For the age groups 13-17-year-olds, that were included later on in the reform, the initial uptake of the services was already high and the increase was less prominent.

The 3.4-fold increase in the absolute number of treatments delivered to Israeli children under NHIL during the study period (from approximately 1.6 million in 2011 to 5.6 million in 2022) indicates a successful implementation of the reform in the oral healthcare system in Israel and a successful transition from entirely private to more public dental care delivery.

The rate at which the number of treatments increased is lower than the rate at which participation increased (3.4 vs. 5.2 fold increase). We assume that the reform contributed to children’s oral health status by providing preventive care and covering existing treatment needs. The decrease in the average number of treatments can also be related to other factors, such as age-related treatment utilization characteristics and the availability and accessibility of the services. Further research of oral health needs and treatment consumption patterns among different age groups is required for a deeper understanding of these findings.

Improvement in the oral health status of Israeli children and trends in treatment coverage and caries morbidity are particularly important for the reform evaluation.

A number of studies conducted several years after the implementation of the reform found a high utilization of publicly funded dental services, showing they became the main treatment framework for children. Thus, a study by the Brookdale Institute conducted in 2013 among parents of 2-11- year-olds eligible for treatment in the reform framework found that 64% of the children visited a dentist in the last year, and about 70% of them received dental care in publicly funded clinics. The impact of the CDCR was particularly significant among Jewish children of low socioeconomic status who used public dental services the most (85%). The percentage of Arab children from low socioeconomic status, using the basket of services offered by the reform, was lower (67%), with smaller proportion of Arab children attending for routine examinations.

According to the Brookdale Institute study, children from a high socioeconomic status visited dental clinics mainly for periodic routine examinations while children from a low socio-economic status visited dental clinics mainly in urgent cases.

Sharhabani et al. [6] found that after the reform started, the frequency of dental examinations among children increased by 26.2%. The increase was even more significant (31.9%) for lower socioeconomic group, which indicated that the reform helped to reduce social gaps between different socioeconomic groups.

In 2016, a national study amongst 1210 6-year-olds found an increase in the treatment component (f / dmft) compared to previous epidemiological dental studies conducted in Israel in this age group [11]. Although no major change in comparison to former surveys was found in caries prevalence, for the first time there was more treated than untreated disease.

Our study findings were in line with previous findings and showed an increase in uptake of dental services by children, especially under age 13.

Preventive dental treatments included in the basket of services are provided with no co-payment, in order to encourage primary prevention, early detection of dental disease, and promote oral health. Our study found that preventive care, in absolute numbers, increased by 400% from 2011 to 2022, which no doubt has a positive influence on children’s health and could be considered as one of the important impacts of the reform.

However, recent studies conducted among children in Israel still found high level of caries. In 2016 a study among 6-year-olds found that 61.7% of children suffered from dental disease. Another study from the Southern region in 2017 found high caries level among kindergarten children, with prevalence of 66.8% and dmft 3.3 [4, 6].

While universal oral health coverage is an important step towards children’s oral health improvement, there are other factors that affect oral health status. Water fluoridation, which is recognized as one of the most effective methods for caries prevention was stopped in Israel in 2014 and has not been reinstated. A recent study shows that with the cessation of water fluoridation, the need for restorative dentistry in children has increased. [12, 13]. Thus, both to prevent caries in the first place and to allow more efficient use of existing dental resources, it is important to reinstate fluoridation as well as to ensure that children who do have existing dental disease receive appropriate dental services.

Other factors such as health literacy, health-related lifestyle and characteristics of care uptake and delivery also affect oral health status [14]. Thus, a study that aimed to explore Israeli-Arab children’s relatively low uptake of publicly funded dental care [15], found numerous possible reasons for low service utilization. Lack of recognition of the importance of preventive care, lack of accessibility and availability of services, various social and cultural factors and economic barriers, which can still exist for large families, or families from low socioeconomic levels (even though the co-payment is small) were among the reasons for the low level of utilization. Therefore, oral health policy makers should consider these factors in the future implementation and improvement of the reform.

According to the aforementioned Brookdale study [16], 64% of 2-11-year-olds children had seen a dentist in the previous 12 months, and of these 71% did so with public funding while 26% were treated privately.

The overall utilization rate of 34% within the dental reform in 2022 is an acceptable outcome (reaches above 50% in certain age groups), considering the current level of caries in Israel and the reports that more than 20% of dental care for children is provided in private settings. Compared to the dental services utilization rate in Israel, dental services utilization rate within the well- established National Health Service (NHS) among children in the UK, was reported in 2019 to be about 60%. A notable decrease in the NHS utilization rate, attributed to the COVID-19 pandemic, was observed in 2020 [17]. This COVID-19 related decrease in utilization is also evident in our findings among Israeli children.

Additional efforts should be undertaken to increase the uptake of publicly funded services, especially by children from lower socioeconomic backgrounds. This can be achieved in accordance with the WHO Global Oral Health Action Plan 2023–2030 [18] guiding principles and objectives, which include parent education, evaluation and removal of barriers, and collaboration with other healthcare providers.

## Conclusion

The aim of this study was to analyze uptake of dental services as a part of dental care NHIL reform. While the utilization of services is satisfying, more efforts are required in order to increase the utilization, especially in low socio-economic groups. Utilization of dental services by different age groups and the effect of treatments provided within the framework of the NHIL reform on children’s oral health need to be continuously assessed in order to evaluate and determine future health policy.

## Data Availability

The datasets analyzed during the current study are not publicly available due to regulatory reasons, but are available from the corresponding author on reasonable request.

## Abbreviations

HMO: Health Maintenance Organization
UHC: Universal health coverage
NHIL: National Health Insurance Law
SDS: School Dental Service
DMFT: Decayed, Missing, Filled Teeth
CDCR: Child Dental Care Reform

## References

[1] Peres MA, Macpherson LMD, Weyant RJ, Daly B, Venturelli R, Mathur MR, et al. Oral diseases: a global public health challenge. Lancet. 2019;394(10194):249–60. 31327369 10.1016/S0140-6736(19)31146-8

[2] Winkelmann J, Listl S, van Ginneken E, Vassallo P, Benzian H. Universal health coverage cannot be universal without oral health. Lancet Public Health. 2023;8(1):e8–10. 36516876 10.1016/S2468-2667(22)00315-2 PMC9810536

[3] Zadik D, Zusman SP, Kelman AM. Caries prevalence in 5- and 12‐year‐old children in Israel. Community Dent Oral Epidemiol. 1992;20(1). 10.1111/j.1600-0528.1992.tb00675.x 1547615

[4] Zusman SP, Ramon T, Natapov L, Kooby E. Dental health of 12-year-olds in Israel-2002. Community Dent Health. 2005;22(3):175–9. 16161882

[5] Natapov L, Sasson A, Zusman SP. Does dental health of 6-year-olds reflect the reform of the Israeli dental care system? Isr J Health Policy Res. 2016;5(1):26. 10.1186/s13584-016-0086-3. 27708769 10.1186/s13584-016-0086-3 PMC5050852

[6] Shahrabani S, Benzion U, Machnes Y, Gal A. The use of dental services for children: implications of the 2010 dental reform in Israel. Health Policy. 2015;119(2):117–26. 10.1016/j.healthpol.2014.11.007. 25465981 10.1016/j.healthpol.2014.11.007

[7] Ministry of Health. Deputy D.G. for Kupot Holim Supervision Circular 12/10, 2010; https://www.health.gov.il/hozer/sbn12_2010.pdf (Hebrew).

[8] Central Bureau of Statistics. https://www.cbs.gov.il/en/subjects/Pages/Live-Births.aspx

[9] Natapov L, Gordon M, Pikovsky V, Kushnir D, Kooby E, Khoury G, et al. Caries Prevalence among five-year-old children examined by the School Dental Service in Israel in 2007. OHDMBSC. 2010;9(1):25–31. https://www.oralhealth.ro/volumes/2010/volume-1/V1-10-6.pdf.

[10] Natapov L, Schwartz D, Herman HD, Markovich DD, Yellon D, Jarallah M, Liphshiz I, Carmeli Y, Karakis I. Risk of SARS-CoV-2 transmission following exposure during dental treatment - A national cohort study. J Dent. 2021Oct;113:103791. 10.1016/j.jdent.2021.103791. 10.1016/j.jdent.2021.103791 PMC8388145 34455018

[11] Natapov L, Sasson A, Zusman SP. Does dental health of 6-year-olds reflect the reform of the Israeli dental care system? Isr J Health Policy Res. 2016;5(1):26. 27708769 10.1186/s13584-016-0086-3 PMC5050852

[12] Tobias G, Mordechai F, Tali C, Yaron B, Beatrice GP, Jonathan M, Harold SC. The effect of community water fluoridation cessation on children’s dental health: a national experience. Isr J Health Policy Res. 2022;11(1):4. 10.1186/s13584-022-00514-z. 35090561 10.1186/s13584-022-00514-z PMC8796457

[13] Levy DH, Sgan-Cohen H, Solomonov M, Shemesh A, Ziv E, Glassberg E, Yavnai N. Association of Nationwide Water Fluoridation, changes in dental care legislation, and caries-related treatment needs: a 9-year record-based cross-sectional study. J Dent. 2023;134:104550. 10.1016/j.jdent.2023.104550. 37196687 10.1016/j.jdent.2023.104550

[14] Natapov L, Dekel D, Pikovsky V, Zusman SP. Dental health of preschool children after two-years of a supervised tooth brushing program in Southern Israel. Isr J Health Policy Res. 2021;10(1):42. 34294158 10.1186/s13584-021-00479-5 PMC8296643

[15] Khatib M, Ashkenazi Y, Loeff Y, Zusman SP, Natapov L. Factors affecting the use of dental services among arab children in Israel: a qualitative study. Isr J Health Policy Res. 2023;12(1):31. 37667386 10.1186/s13584-023-00579-4 PMC10476419

[16] Ashkenazi Y, Yankellevich A, Zusman SP, Natapov L, Hebrew.). 2016. https://brookdale.jdc.org.il/en/publication/patterns-utilization-experiences-children-dental-care-following-reform-dental-care-israel/

[17] Two-thirds of children did not see an NHS dentist last year. BDJ Team. 2021;8:4. 10.1038/s41407-021-0562-7. 10.1038/s41407-021-0562-7

[18] World Health Organization. WHO Global Oral Health Action Plan 2023–2030. 2022. https://cdn.who.int/media/docs/default-source/ncds/mnd/eb152-draft-global-oral-health-action-plan.pdf

